# Translating a National Laboratory Strategic Plan into action through SLMTA in a district hospital laboratory in Botswana

**DOI:** 10.4102/ajlm.v3i2.209

**Published:** 2014-11-03

**Authors:** Keoratile Ntshambiwa, Winnie Ntabe-Jagwer, Chandapiwa Kefilwe, Fredrick Samuel, Sikhulile Moyo

**Affiliations:** 1 Sekgoma Memorial Hospital, Ministry of Health, Botswana; 2 Botswana-Harvard AIDS Institute Partnership and Botswana-Harvard HIV Reference Laboratory, Princess Marina Hospital, Botswana

## Abstract

**Background:**

The Ministry of Health (MOH) of Botswana adopted Strengthening Laboratory Management Toward Accreditation (SLMTA), a structured quality improvement programme, as a key tool for the implementation of quality management systems in its public health laboratories. Coupled with focused mentorship, this programme aimed to help MOH achieve the goals of the National Laboratory Strategic Plan to provide quality and timely clinical diagnoses.

**Objectives:**

This article describes the impact of implementing SLMTA in Sekgoma Memorial Hospital Laboratory (SMHL) in Serowe, Botswana.

**Methods:**

SLMTA implementation in SMHL included trainings, improvement projects, site visits and focused mentorship. To measure progress, audits using the Stepwise Laboratory Quality Improvement Process Towards Accreditation (SLIPTA) checklist were conducted at baseline and exit of the programme, with scores corresponding to a zero- to five-star scale. Turnaround times, customer satisfaction, and several other health service indicators were tracked.

**Results:**

The laboratory scored 53% (zero stars) at the baseline audit and 80% (three stars) at exit. Nearly three years later, the laboratory scored 85% (four stars) in an official audit conducted by the African Society for Laboratory Medicine. Turnaround times became shorter after SLMTA implementation, with reductions ranging 19% to 52%; overall patient satisfaction increased from 56% to 73%; and clinician satisfaction increased from 41% to 72%. Improvements in inventory management led to decreases in discarded reagents, reducing losses from US $18 000 in 2011 to $40 in 2013.

**Conclusion:**

The SLMTA programme contributed to enhanced performance of the laboratory, which in turn yielded potential positive impacts for patient care at the hospital.

## Introduction

Laboratory medicine plays a critical role in healthcare and public health,^1,2,3^ and expanding laboratory capacity has been a focus of funding partners such as the US President’s Emergency Plan for AIDS Relief (PEPFAR).^4^ Implementation of laboratory quality management systems (QMS) and accreditation to international standards contribute to increased accuracy of diagnoses and improvement in patient management.^3^ Although considerable resources have been invested in recent years for the improvement of laboratory systems in resource-limited settings, lack of access to reliable laboratory testing still remains a barrier to effective healthcare and treatment for diseases, including tuberculosis, HIV and malaria.^5,6,7^ Amongst the numerous challenges identified are the lack of national standards and policies specifically addressing QMS.^2,3^ In 2008, the World Health Organization’s (WHO) Maputo Declaration^8^ called for the adoption of national laboratory strategic plans; Nkengasong et al. concurred, arguing that such plans are one of the essential requirements for laboratory capacity building.^9^

The Ministry of Health (MOH) in Botswana recognises the need for a collaborative and coordinated effort to raise the standards of its national laboratories. However, over the years there has been slow progress in implementing QMS. Previous training of healthcare workers has focused on general management topics rather than identifying tangible tasks to bring about change, making the training difficult to apply in the laboratory.^10^ In 2009, the MOH developed a Health Sector Laboratory Strategic Plan for 2009–2014, in which laboratory accreditation was a stated goal: to have four district-level laboratories accredited by 2013 and two national-level laboratories accredited by 2014. It became apparent that a radical shift in strategy would be required in order to realise these ambitious goals. To spearhead the MOH’s National Laboratory Strategic Plan, Strengthening Laboratory Management Toward Accreditation (SLMTA), a structured quality improvement programme, was selected to assist in the development of QMS within healthcare facilities.^10,11^ Since its launch in 2009, SLMTA has demonstrated success in many countries^12^ because of its task-oriented measureable design.

Sekgoma Memorial Hospital Laboratory (SMHL), a district laboratory of the Sekgoma Memorial Hospital, was selected by the MOH as one of eight laboratories to participate in the first SLMTA training cohort. Sekgoma Memorial Hospital is a 350-bed facility located in Serowe, a trade and commerce centre located in Central District in eastern Botswana. SMHL has eight units: chemistry, haematology, microbiology, CD4, viral load, blood bank, serology and reception. Patient samples for laboratory testing originate from the hospital wards, from 18 clinics within the hospital’s catchment area, and from outpatient services. On average, approximately 100 samples are received daily for chemistry, CD4 and haematology testing; 150 samples for viral load testing; and 30 samples for microbiology testing. This article describes the implementation and results of the SLMTA training and mentorship programme at SMHL.

## Research methods and design

The Botswana SLMTA training programme was conducted in accordance with the standard SLMTA implementation model.^11^ Training consisted of a series of three workshops, each lasting four days, held in August 2010, November 2010 and February 2011. Attendees from SMHL included the laboratory manager, the quality officer and two section supervisors. At each workshop, attendees selected improvement projects to implement in the laboratory based on the SLMTA modules presented. Two supervisory visits of one day each were conducted by the three SLMTA trainers following each workshop.

Programme performance was measured by audits conducted before (baseline, July 2010) and after (exit, November 2011) SLMTA implementation using the WHO’s Regional Office for Africa (AFRO) Stepwise Laboratory Quality Improvement Process Towards Accreditation (SLIPTA) checklist.^13^ SLIPTA is an accreditation preparation framework, which measures and rewards the incremental progress of implementing QMS requirements using a comprehensive audit checklist addressing the 12 Quality System Essentials of the laboratory.^14^ The SLIPTA checklist and scoring system rate laboratory quality on a scale of zero to five stars; a rating of five stars signifies that the laboratory is ready to seek international accreditation. In-country auditors trained by A Global Healthcare Public Foundation, an independent external contractor based in Kenya, conducted the baseline and exit audits. In August 2014, SMHL received an official WHO AFRO SLIPTA audit by the African Society for Laboratory Medicine.

From April to June 2011, SMHL received focused mentorship from the Botswana Bureau of Standards (BOBS) to augment the SLMTA training. The BOBS mentorship programme consisted of monthly visits, each lasting one week, during the three-month mentoring period. The visits included training on the International Organization for Standardization (ISO) 15189 standard and QMS documentation, with a focus on the management requirements of ISO 15189. All laboratory staff members participated in the training sessions, whilst key staff members received additional mentorship on essential documentation of the QMS.

The laboratory developed and implemented four overarching improvement projects to address deficiencies identified during the baseline audit. A Plan-Do-Check-Act (PDCA) cycle was implemented in order to facilitate continuous quality improvements, with past results driving future activities. Progress of improvement projects was monitored using audit items selected from the SLIPTA checklist, which were tracked and recorded. Below we describe the improvement projects implemented: reducing sample turnaround time; increasing customer satisfaction; the ‘6S’ project; and improving specimen management and documentation.

### Improvement project 1: Reducing sample turnaround time

In clinical laboratories, turnaround time is the total elapsed time from when a test is ordered to when the results are verified and released. A target turnaround time of two hours was established for haematology, chemistry and cerebrospinal fluid (CSF) tests; a target of one hour was established for pregnancy tests. To assess this improvement project, data were obtained from the laboratory computer system, the Integrated Patient Management System, on a monthly basis. Average turnaround time for tests was analysed for two 6-month periods: April to September 2011 and October 2011 to March 2012. Turnaround time data from before SLMTA implementation were not available.

### Improvement project 2: Increasing patient and clinician satisfaction

Customer satisfaction surveys are mandated by MOH guidelines. Separate questionnaires were administered for both patients and clinicians once a year in 2011, 2012 and 2013. The questionnaires utilised a rating system of ‘poor’, ‘fair’, ‘good’ and ‘very good’, with the latter two responses interpreted as ‘satisfied’ for purposes of analysis. The patient questionnaire asked patients to evaluate the laboratory reception, the staff members’ ability to answer questions, staff behaviours and attitudes, staff availability, the waiting time for test results, and overall satisfaction. Patient questionnaires were distributed to patients every morning for a period of one month at the end of each year in 2011, 2012 and 2013. The clinician questionnaire asked staff to rate hospital services, turnaround times, and overall satisfaction; evaluate the attitudes of laboratory staff; and suggest ways to respond to enquiries. Twenty doctors and nurses in each of the 10 hospital wards completed the clinician questionnaires for each year of evaluation.

### Improvement project 3: ‘6S’

The SLMTA team implemented ‘6S’ (sorting, straightening, shining, standardising, sustaining and safety) in order to improve storage space and reduce expired products. An action plan was developed for each issue identified. Wastage generated from discarded products as a result of expiry was tracked and their value calculated for fiscal years (FY) 2011 and 2013.

### Improvement project 4: Improving specimen management and document control

To improve specimen management, missing and deficient essential documents were identified based on the SLIPTA checklist and ISO 15189 requirements. Standard operating procedures (SOPs) were created and revised, including system procedures, safety procedures and technical procedures; staff were then trained on the relevant SOPs. Signs with directions were placed in all testing areas so as to facilitate movement of staff and clients throughout the facility. Selected hospital drivers attended a half-day workshop of presentations and simulation exercises, where they were trained on safe specimen handling, proper transportation and timely result distribution.

## Results

The laboratory audit score improved from zero stars (53%) at baseline in July 2010 to three stars (80%) at the exit audit in November 2011. At the official WHO AFRO SLIPTA audit in August 2014, the laboratory received four stars (85%).

**FIGURE 1:**
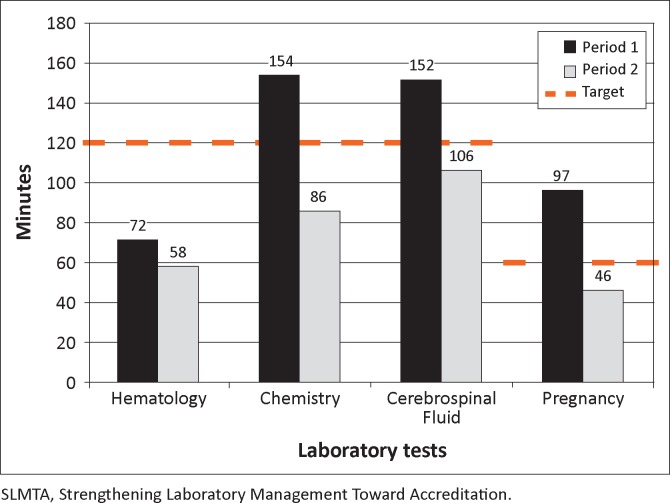
Average turnaround time (minutes) for laboratory tests in April to September 2011 (period 1) and October 2011 to March 2012 (period 2).

Turnaround times decreased for all tests monitored: 19% reduction for haematology tests, 44% for chemistry tests, 30% for CSF analyses and 52% for pregnancy tests (Figure 1). Pre-set targets of one hour for pregnancy tests and two hours for the remaining tests were all met.

Overall patient satisfaction increased from 56% in 2011 to 85% in 2012, then decreased to 73% in 2013 (Figure 2a). This pattern was consistent in each of the individual questions. Clinician satisfaction increased steadily from 41% in 2011 to 64% in 2012 and 72% in 2013 (Figure 2b), with steady improvements in all areas.

**FIGURE 2a:**
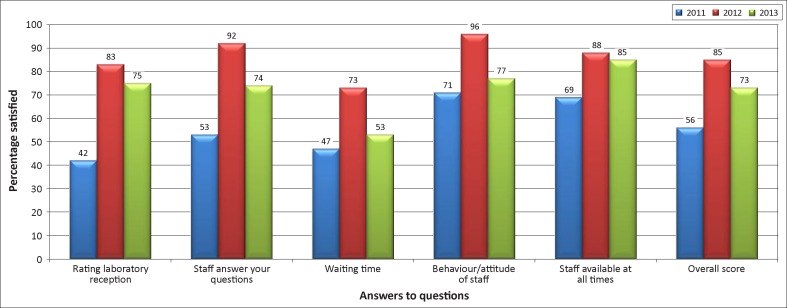
Percentage of patients who reported satisfaction with selected services (2011 to 2013).

**FIGURE 2b:**
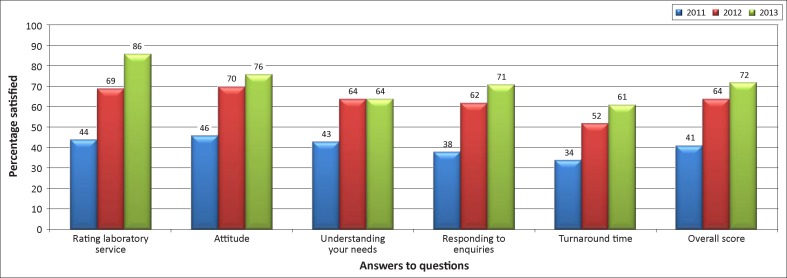
Percentage of clinicians who reported satisfaction with selected services (2011 to 2013).

The ‘6S’ improvement project led to the reorganisation of workstations and removal of unused items, which resulted in the creation of space and the alleviation of staff overcrowding. Storage shelves were labelled with contents and the storeroom was cleaned and reorganised; all expired items were removed. Systematic tracking of reagent expiration dates and improved inventory management led to a decrease in discarded reagents, with a subsequent drop in laboratory losses from P128 000 (approximately $18 000) in FY2011 to P280 (approximately $40) in FY2013.

Improved sample management and documentation revealed problems with specimen collection, especially a high sample rejection rate caused by poor phlebotomy. As a result, a Specimen Collection Manual was developed and distributed to all clinics and wards in the hospital and guidance was provided. A total of 154 SOPs were developed and implemented, including management and technical documents for specimen receipt and results dispatch. Long turnaround times during lunch hours were reduced by implementing an improved mid-day staff coverage system. A sign-in book for sample delivery was initiated in order to reduce delays in the transport of samples from the laboratory reception to the benches. All urgent samples were tagged with a white sticker on the cap to make their priority visible.

## Discussion

Implementation of SLMTA and targeted mentorship enabled SMHL to develop an effective QMS, including mechanisms to monitor critical aspects of laboratory service in pre-analytical, analytical and post-analytical processes. This resulted in rapid and measurable changes as evidenced by the jump from zero stars at the baseline audit to three stars at the exit audit. These improvements were sustained and further increased, with the laboratory reaching four stars at the official WHO SLIPTA audit nearly three years later.

Reduction of turnaround time and improved customer satisfaction after completion of the SLMTA programme also indicated positive and sustained impact on patient care. The decrease in turnaround time for haematology occurred despite an increase in testing volume; this was because of a combination of reduced equipment downtime as a result of effective preventive maintenance of analysers and reduced stock-outs from the introduction of an effective inventory management system. SLMTA training enabled the laboratory staff to take on the daily challenges of increased volume that could otherwise strain the laboratory system.

One important lesson learned was that staff motivation and management buy-in are critical for the success of the SLMTA programme. Involving management in the early stages of SLMTA planning and periodic structured updates facilitated their understanding of QMS and built consensus on the collaborative role of each department in achieving the goals of the National Laboratory Strategic Plan. Productive and frequent discussions between clinicians and laboratory staff paved the road to success and became a vehicle for translating strategic plans to action.

The SLMTA programme brought about a cultural shift at SMHL by providing the staff with a step-by-step process to break down tasks into actionable items with the common goal of providing high-quality laboratory services. The focus on continuous quality improvement ensures that the gains are maintained in a practical way, with active involvement of management and clinicians in identifying and addressing remaining challenges.

Staff motivation was also noted as a key driver to implementing QMS. For instance, immediate changes in the organisation and utilisation of space as a result of the ‘6S’ project improved staff morale, largely because staff members were involved directly in the results-oriented process. This immediate transformation encouraged and motivated the staff to find other solutions using existing resources. Improvement projects that were identified and planned during the SLMTA training stimulated creativity, ownership and action amongst laboratory and hospital staff; and were a great asset to the quality improvement programmes at SMHL. It was noted by management that the more the staff were trained on SLMTA, the more enthusiastic and confident they became, thus enhancing the working relationship between staff and clients.

In addition to SLIPTA scores and stars, monitoring quality indicators such as turnaround time and customer satisfaction was valuable for assessing the laboratory’s success and identifying areas needing improvement. For example, customer satisfaction surveys alerted laboratory staff to declining patient satisfaction with waiting times from 2012 to 2013. After brainstorming sessions, the team concluded that this decline may have been caused by a failure to communicate to patients about the newly-introduced practice of prioritising services to patients needing urgent assistance such as pregnant women, the elderly and the disabled. Armed with results from the survey, laboratory staff were able to address the problem head-on, by improving communication with patients to ensure that they understood the purpose of the new triage system.

### Limitations of the study

We acknowledge some limitations associated with this work. Primarily, data for turnaround time and customer satisfaction were not collected before SLMTA implementation, preventing a pre-/post-SLMTA comparison. Thus, our results are likely an underestimate of the true effect of SLMTA implementation.

### Conclusion

The Botswana MOH has made great strides in its drive toward continuous quality improvement in public-sector laboratories by adopting the SLMTA programme and focused mentorship as tools to help translate the National Laboratory Strategic Plan into action. Whilst tuberculosis, HIV, malaria and other diseases remain a challenge in Botswana, the achievement of quality, timely and accurate laboratory results will play an important role in reducing the incidence of infections and improving treatment outcomes. At SMHL, the SLMTA programme has been essential for the strengthening of laboratory management systems and has helped lay a firm foundation for further advancements in patient care.
